# On the Use of Multi-Step Dies for Improving the Performance against Hydrogen Embrittlement of Cold Drawn Prestressing Steel Wires

**DOI:** 10.3390/ma15249085

**Published:** 2022-12-19

**Authors:** Jesús Toribio, Miguel Lorenzo

**Affiliations:** Fracture & Structural Integrity Research Group, University of Salamanca, E.P.S., Campus Viriato, Avda, Requejo 33, 49002 Zamora, Spain

**Keywords:** prestressing steel wires, cold drawing, cold drawn pearlitic steel wires, drawing die, die design, residual stresses, plastic strains, hydrogen embrittlement

## Abstract

The main cause of in-service failure of cold drawn wires in aggressive environments is hydrogen embrittlement (HE). The non-uniform plastic strains and residual stresses generated after cold drawing play a significant role in the matter of HE susceptibility of prestressing steels. In this paper, a new and innovative design of the drawing scheme is developed, geared towards the reduction in both manufacturing-induced residual stresses and plastic strains. To achieve this goal, three innovative cold drawing chains (consisting in diverse multi-step dies where multiple diameter reductions are progressively carried out in a single die) are numerically simulated by the finite element (FE) method. From the residual stress and plastic strain fields revealed from FE numerical simulations, hydrogen accumulation for diverse times of exposure is obtained by means of FE simulations of the hydrogen diffusion assisted by stress and strains. Thus, an estimation of the HE susceptibility of the cold drawn wires after each process was obtained. Results reveal that cold drawn wire using multi-step dies exhibits lower stress and strain states nearby the wire surface. This reduction causes a decrease in the hydrogen concentration at the prospective damage zones, thereby improving the performance of the prestressing steel wires in hydrogenating environments promoting HE. Thus, the optimal wire drawing process design is carried out using special dies with several reductions per die.

## 1. Introduction

The use of cold-drawn prestressing steel wires is widely extended in civil engineering as constituents of prestressed concrete structures. These steels exhibit a high susceptibility to the deleterious action of hydrogenating environment in the form of hydrogen embrittlement (HE) [1,2,3,4,5]. Mainly, two effects are produced in the wires after wire drawing: (i) an improvement of the mechanical resistance [6] and (ii) the generation of a non-uniform plastic strain field causing a residual stress field in the wire after drawing [7]. Residual stresses generated after wire drawing play a key role in the structural integrity assessment of prestressing steels during their life in service since these stresses modify the fracture and fatigue behaviour of prestressing steel wires [8,9,10,11]. For this reason, many studies are focused on the analysis of the residual stress state generated in a wire drawn during the material conforming process, either experimentally by means of diffraction techniques, such as X-ray or neutron diffraction [12,13,14,15], or numerically by means of finite element (FE) simulation [16,17,18]. Some studies analyze how the geometry of the die influence on the residual stress state, focusing on die geometric parameters, such as the inlet reduction angle, the bearing length, or the reduction in area [19,20,21,22,23]. However, only some analyses [19,20] have been focused on diminishing residual stress in a cold drawn wire by modifying the drawing die geometry in a single-step drawing process. Briefly, one of these die geometry modifications (named protrusion die [20]) consists of a soft diameter reduction after the main reduction performed either in an additional die or in the same die, which leads to an improvement of the wire smoothness after the conforming process [24].

The aim of the aforesaid studies [19,20,24] was the improvement of the mechanical behaviour of such wires in an inert environment (non-aggressive). Thus, only the axial residual stress was analyzed, i.e., the component of the stress tensor superposed to the externally applied stress, which is, obviously, axial because the wire is subjected only to tension loading in service. In spite of these simple analyses, previous studies [7,25] demonstrated the key role of other mechanical parameters in hydrogenating environments causing HE, namely the hydrostatic stress and the equivalent plastic strain. For this reason, it would be desirable to include both the hydrostatic stresses and the plastic strains in the analysis of the advantages of optimized wire drawing processes by modifying the die geometry. Studies related with this topic [26,27] showed the influence of the parameters that define the geometry of a drawing die (bearing length and inlet die angle) on the HE susceptibility of cold-drawn prestressing steel wires.

Within this framework, the aim of this study is to estimate the influence of the residual stress and strain state generated in wires drawn with modified dies on the behaviour against HE of such wires. To achieve this goal, three new design cold drawing chains (consisting in diverse multi-step dies where multiple diameter reductions are progressively carried out in a single die) are numerically simulated by FE and, lately, compared with a real cold drawing chain (a conventional multi-step process consisting in diverse diameter reductions, each one of them performed in a single die). Thus, considering the residual stress and plastic strain fields given by FE numerical simulations, an estimation of the HE susceptibility of the cold drawn wires after each process can be obtained in terms of hydrogen accumulation for diverse times of exposure (including the steady state) to a hydrogenating environment. This way, the hydrogen concentration field was revealed by means of FE numerical simulation of the hydrogen diffusion assisted by stress and strain states. The final aim of the new multi-step dies is reducing both the residual stress and the plastic strain states caused in the wire by cold drawing and thus improving the mechanical performance of these structural components.

## 2. Materials and Methods

### 2.1. Design of Multi-Step Drawing Dies

**Figure 1:**
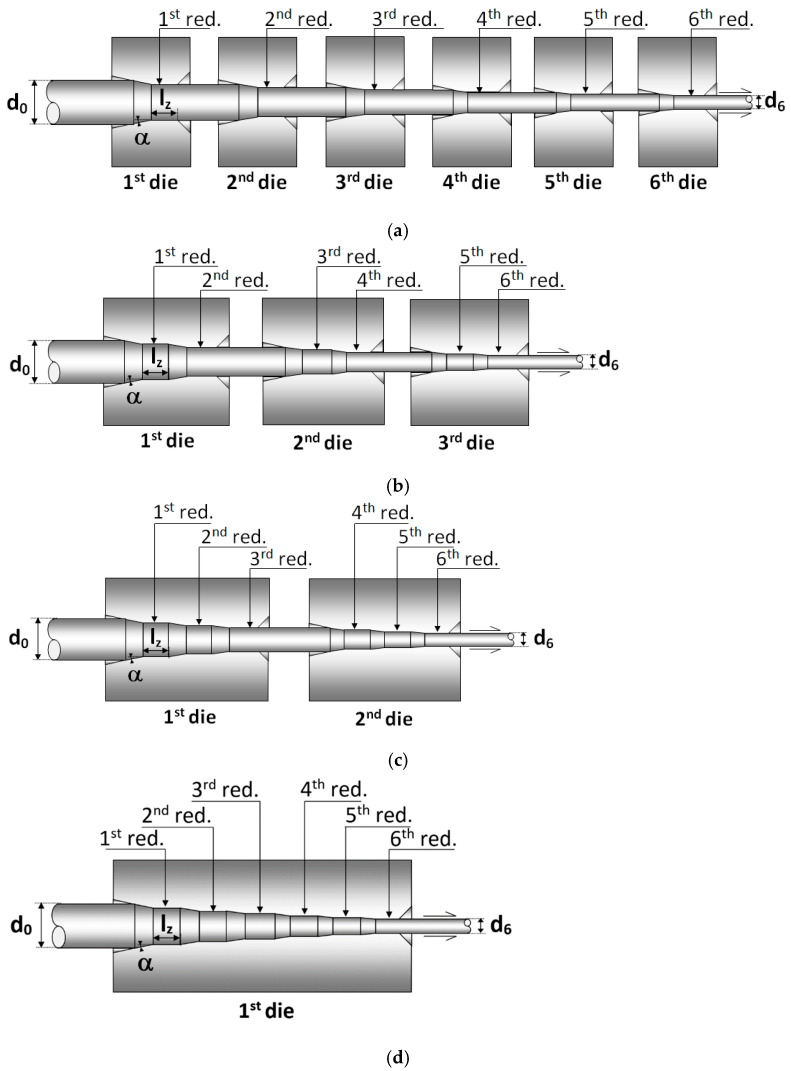
Scheme of simulated wire drawing processes: (**a**) one reduction per die (*conventional drawing*), (**b**) two reductions per die, (**c**) three reductions per die, and (**d**) six reductions per die.

Wire drawing consists of a huge decrease in the wire diameter frequently carried out in progressive reduction steps [6,7] each one of them corresponding to a die. Nowadays, in the case of cold drawn prestressing steels, 6 or 7 drawing steps are commonly used [6] in drawing industry (Figure 1a). This configuration of the drawing process requires large drawing machines. In this paper, the improvements of using a new design of the drawing die are analyzed in which several diameter reductions are applied in the same die. Four different drawing processes were simulated by means of FE with a commercial code (MSC.Marc): (i) *process A*, the conventional wire drawing process (Figure 1a) composed by 6 diameter reductions developed in 6 dies, i.e., one reduction per die, (ii) *process B*, a wire drawing process where the six reductions were performed in 3 dies (Figure 1b), i.e., two reductions per die, (iii) *process C*, a wire drawing process considering six reductions in 2 dies (Figure 1c) that implies three reductions per die, and finally, (iv) *process D*, a wire drawing chain considering six reductions in just one die (Figure 1d).

**Figure 2:**
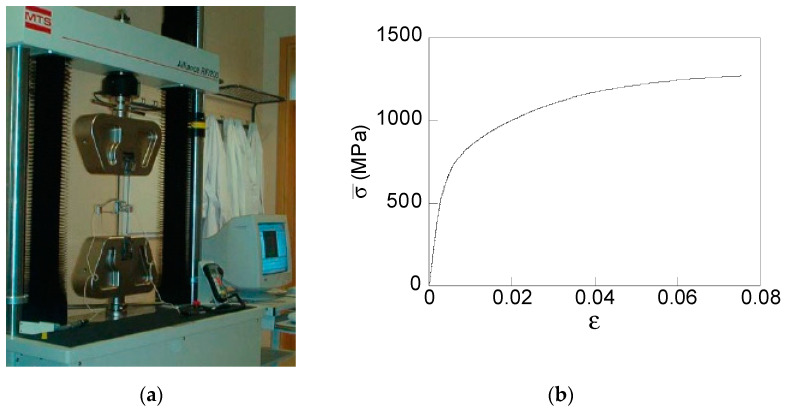
Conventional tension test: (**a**) experimental set-up and (**b**) experimental stress–strain curve of the raw material before cold drawing.

To reveal the advantages of the proposed multi-step dies, a real (commercial) cold drawing chain was considered. The chemical composition of the basic raw material, a hot-rolled pearlitic steel, was the following: 0.800% C, 0.690% Mn, 0.230% Si, 0.012% P, 0.009% S, 0.004% Al, 0.265% Cr, and 0.060% V. The mechanical properties of the pearlitic steel considered in FE simulations were obtained by testing samples of 300 mm length of hot rolled bars corresponding to a real commercial wire drawing chain. Slim specimens were tested as received from the wire drawing company, i.e., without any treatment, in order avoid a modification of the manufacturing-induced residual stress. Conventional tension test up to fracture under a constant displacement rate of 2 mm/min in a universal test machine (MTS KN200) were carried out (Figure 2a). Sample displacements were recorded with two MTS axial extensometers (25 and 50 mm of gage length) placed at the specimen. From the test, the material true stress–strain curve was obtained (Figure 2b), revealing the material properties: Young’s modulus *E* = 194 GPa and 0.2% offset yield strength *σ*_Y_ = 720 MPa. 

In numerical simulations, an elastoplastic solid was selected as a constitutive model with von Mises yield surface and isotropic strain-hardening according to the material true stress true–strain curve shown in Figure 2. The four drawing processes apply on the same initial wire (a hot rolled bar of *d*_0_ = 12 mm) the same wire reductions according to the sequence used in the industrial wire process: *d*_0_ = 12.00 mm, *d*_1_ = 10.80 mm, *d*_2_ = 9.75 mm, *d*_3_ = 8.90 mm, *d*_4_ = 8.15 mm, *d*_5_ = 7.50 mm, and *d*_6_ = 7.00 mm. Thus, at the end of the processes, a cold drawn wire with the same final diameter (*d*_6_ = 7 mm) is obtained. The die geometry parameters (cf. Figure 1a), inlet die angle (α) and bearing length (*l*_z_) were chosen from the data available of a real commercial wire drawing chain used in previous studies [7,26]: inlet die angle, α = 7° and die bearing length, *l*_z_ = *d*_0_. The same die parameters were considered for each step of the two *newly proposed* wire drawing processes B and C (Figure 1b,c). However, in the case of the process D (six reductions per die, i.e., the whole drawing chain, Figure 1d), necking appears during drawing when conventional inlet die angle (α = 7°) is used. Therefore, to achieve the whole drawing *in only one die*, the value of the inlet die angle was reduced up to α = 2° at each one of the six drawing reductions. At the end of the reduction zones, the same die bearing length was considered (*l*_z_ = *d*_0_) as in the other drawing processes. 

Taking into account the revolute symmetry of both wire and die, the axisymmetric formulation is adequate for FE simulations. To obtain the desired wire dimensions after drawing, dies must not undergo high displacements or permanent strains during the drawing process. Consequently, materials with a high stiffness and high strength, such as ceramic materials (for instance, wolfram carbide, CW), are commonly used. Thus, a fine approach for modeling this component in FE simulations is to consider dies as rigid bodies. Wire was considered as a deformable body. A non-uniform mesh in the radial direction of the wire was applied using four node quadrilateral elements. Thus, the size of the elements is progressively decreased from the wire center up to the wire surface where the contact between die and wire is produced during drawing. Thus, a fine meshing nearby the wire surface is obtained without significantly increasing the total number of elements. Diverse meshes were considered to ensure an acceptable mesh convergence in results, and a final mesh of 3381 nodes and 3200 elements was used in FE simulations. The boundary conditions applied are the following: on one hand, a null displacement of the nodes placed at the symmetry axis of the wire due to axisymmetric assumption and, on the other hand, a constantly growing axial displacement that was applied to the nodes placed at the front extreme of the wire. The final displacement was selected in such a way that the whole wire passes the last die of the drawing chain. Elastoplastic large deformation–large strain calculations were performed using a general-purpose finite element code (MSC.Marc) with updated lagrangian formulation. 

### 2.2. Hydrogen Diffusion Assisted by Stress and Strain

HE is a complex process, which develops in several stages [28,29,30]: (i) the molecular hydrogen adsorption in the material surface from the harsh environment, (ii) the dissociation of the hydrogen molecule, (iii) the atomic hydrogen absorption into the material, (iv) the hydrogen transport by diffusion through the metallic material lattice towards prospective micro-damage zones [31] where hydrogen is accumulated [32] until reaching a critical concentration linked with the hydrogen microstructural damage. The stage that rules the HE process is the hydrogen diffusion, which is influenced by the stress and strain states. According to the well-known model of hydrogen diffusion assisted by stress and strains, hydrogen diffuses from the wire surface toward the wire core in terms of the gradient of hydrogen concentration, the inward gradient of hydrostatic stress (*σ*) and the inward gradient of hydrogen solubility (*K*_sε_) [28,29,30,31,32,33]:

(1) $$J=-D({\bar{\varepsilon }}_{P})\left\{\nabla C-C\left[\frac{V_{H}}{RT}\nabla \sigma +\frac{\nabla K_{S\varepsilon }({\bar{\varepsilon }}_{P})}{K_{S\varepsilon }({\bar{\varepsilon }}_{P})}\right]\right\},$$

**J** being the hydrogen flux, *D* the diffusion constant, *C* the hydrogen concentration, *R* the molar gas constant, *V*_H_ the partial volume of hydrogen, *T* the absolute temperature, and *K*_sε_ the hydrogen solubility. According to different studies [7,28], hydrogen solubility increases with the equivalent plastic strain, and commonly, the following linear relation is used, *K*_sε_ = 1 + 4 ε_p_, cf. [7,28].

The hydrogen diffusion equation can be expressed as a second-order differential partial equation applying the matter conservation law and the Gauss–Ostrogradsky theorem, resulting:

(2) $$\frac{\partial C}{\partial t}=\nabla \cdot \left[D\nabla C-DC\left(\frac{V_{H}}{RT}\nabla \sigma +\frac{\nabla K_{S\varepsilon }({\bar{\varepsilon }}_{P})}{K_{S\varepsilon }({\bar{\varepsilon }}_{P})}\right)\right].$$

**Figure 3:**
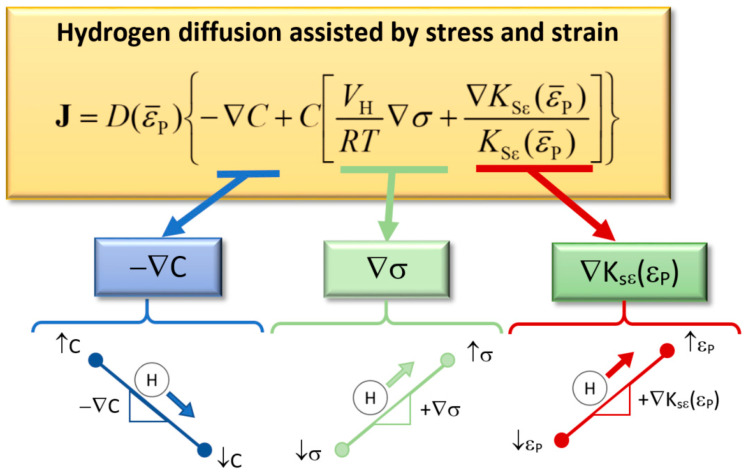
Scheme of the influence on hydrogen diffusion of the driven forces according to the model of diffusion assisted by stress and strain.

Thus, from Equation (2), the effect on hydrogen diffusion of the three driven forces is easily understandable. Hydrogen diffuses toward the inner points due to: (i) the negative gradient of hydrogen concentration (similar to the Fick’s law); (ii) the positive gradient of hydrostatic stress; and (iii) the positive gradient of hydrogen solubility, which is proportional to the gradient of equivalent plastic strain in the case of linear relationship and four times its value if the function *K*_sε_ = 1 + 4 ε_p_ is used [7,28]). For the sake of clarity, Figure 3 shows a scheme of the driving forces for hydrogen diffusion assisted by stress and strains.

This way, the analysis of the gradient of equivalent plastic strain will lead to the equivalent results of those obtained from the analysis of hydrogen solubility gradient considering the linear dependence of the latter variable on an equivalent plastic strain. 

In the present study, the depth from the wire surface is represented by the variable *x* = *d*_i_/2 − *r*. Thus, the variable of interest is the inward gradient ∂/∂*x* (i.e., from wire surface toward the wire core, and consequently, the following relationship must be satisfied ∂/∂*x* = −∂/∂*r*). This way, hydrogen diffusion is developed from the wire skin to the prospective damage locations according to the inward gradient ∂/∂*x*. Taking into account such processes, the main attention must be paid on the wire surface Γ (*r* = 3.5 mm) and its respective inward gradient (∂/∂*x*)_Γ_.

The concentration of hydrogen for long times of exposure, *C*_eq_ (equilibrium state reached for infinite time) can be obtained by the following steady-state solution of differential Equation (2) in a well-known Maxwell–Boltzman type:

(3) $$C_{\mathrm{eq}}=C_{0}K_{S\varepsilon }({\bar{\varepsilon }}_{P})\exp\left[\frac{V_{H}}{RT}\nabla \sigma \right],$$

where *C*_0_ is the equilibrium hydrogen concentration for the material free of stress and strain.

The radial distributions of residual stress and plastic strain obtained in numerical simulations are similar for any cross section of the wire in the axial direction (∂/∂*z* = 0, considering *z* as the axial coordinate). Therefore, main variations in the stress and strain field only appear in the radial direction of the wire. Thus, the analysis of the hydrogen transport by diffusion can be solely focused on the afore-said radial direction. Therefore, a one-dimensional (1D) axisymmetric approach in the radial direction of the wire is adequate for estimating metal hydrogenation. Thus, a FE numerical simulation of the axisymmetric boundary-value problem of stress–strain-affected diffusion (Equation (2)) was implemented in a general purpose mathematical software (MathCAD 14). For the sake of simplicity, the trial and weighting functions used are the element shape functions *N*_e_(*r*) applying the Galerkin method [30]. In addition, such functions were also considered for the approximation of hydrostatic stress *σ*(*r*) and equivalent plastic strain ε*_P_*(*r*) fields as:

(4) $$\sigma (r)=\sum \sigma_{j}N_{j}(r),\varepsilon_{P}(r)=\sum \varepsilon_{\mathrm{Pj}}N_{j}(r),$$

where *j* = 1,…, *M* corresponds to the number of node of the FE mesh, *M* being the total number of nodes.

This way, by applying the weak form of the weighted residual statement of the problem, a system of ordinary differential equations is obtained according to the following expression in terms of the time-dependent FE nodal concentration values *C*_j_ (*t*):

(5) $$\left[M_{\mathrm{ij}}\right]\left\{\frac{dC_{j}}{dt}\right\}+\left[K_{\mathrm{ij}}\right]\left\{C_{j}\right\}=\left\{F_{i}\right\}\;(i,j\;=\;1,\ldots ,M),$$

In this equation, the components of the element matrices [...] and the vector columns {...} are obtained as follows:

(6) $$M_{\mathrm{ij}}=\int_{V}N_{i}N_{j}dV,$$

(7) $$K_{\mathrm{ij}}=\int_{}D\left(\varepsilon_{P}\right)\left\{\nabla N_{i}\nabla N_{j}-\left[\left(\frac{V_{H}}{RT}\nabla \sigma +\frac{\nabla K_{S\varepsilon }\left(\varepsilon_{P}\right)}{K_{S\varepsilon }\left(\varepsilon_{P}\right)}\right)\cdot \nabla N_{i}\right]N_{j}\right\}dV,$$

(8) $$F_{i}=-J_{s}\int_{S_{f}}N_{i}dS,$$

considering the flux of hydrogen *J*_S_ applied on the surface zone *S*_f_. 

Thus, the solution of the system of first-order differential Equation (5) may be obtained by programming the time-marching numerical scheme proposed for diffusion-type equations [34]. Accordingly, the nodal concentration values at the initial instant (*C*_m−1_) of the *m* time interval [*t*_m−1_,*t*_m_] and at the end of such an interval (*C*_m_) are linked according to:

(9) $$\left(C_{m}-C_{m-1}\right)\left(M+\theta \;\Delta t\;K\right)/\Delta t+KC_{m-1}=F$$

where Δ*t* = *t*_m_ − *t*_m−1_. The parameter θ must be selected to ensure the stability of this time-marching scheme. Thus, this procedure of time integration is unconditionally stable if θ is included in the following interval, θ ∈ [0.5, 1] [34]. The array *C*_0_ for the first-time interval (at *m* = 1) corresponds to the prescribed initial conditions of the analyzed problem. Afterward, the values for *C*_m_ are obtained by solving the following equation in matrix form.

(10) $$C_{m}=C_{m-1}+{\left(M+\theta \;\Delta t\;K\right)}^{-1}\left(F-KC_{m-1}\right)\Delta t$$

which applying suitable algorithms of matrix inversion provides the hydrogen distribution for any time during exposure to the hydrogenating environment. 

For the sake of simplicity, meshing considered in the FE simulation of wire drawing was used for simulating the hydrogen diffusion assisted by the stress and strain process assuming linear trial functions for both space and time variables. The parameters involved in the simulations were selected as follows: fixed temperature, *T* = 298 K; partial molar volume of hydrogen for iron-based alloys, *V*_H_ = 2 cm^3^/mol [35]. Hydrogen diffusivity, *D*, is very sensitive to changes in chemical composition, microstructure, and cumulative plastic strain. This way, this parameter at room temperature is approximately 10^−12^ m^2^/s and even lower [36] for heavily drawn wires with a high density of microstructural defects, such as dislocations, strained grain boundaries in the pearlitic lamellae, etc. In this study, the hydrogen diffusivity for the hot rolled bar A0 and for the commercial prestressing steel wires A6 were taken from previous studies as follows: *D*^(0)^ = 6.6 × 10^−11^ m^2^/s [37] and *D*^(6)^ = 4.99 × 10^−12^ m^2^/s [36], respectively. Notice that the superindex (*i*) indicates the wire drawing step.

## 3. Results

### 3.1. Stress and Strain States

**Figure 4:**
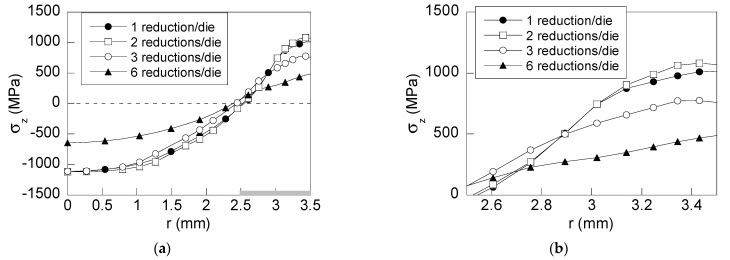
Radial distribution of axial stress: (**a**) general view; (**b**) detailed view of the important near-surface region of the wire.

**Figure 5:**
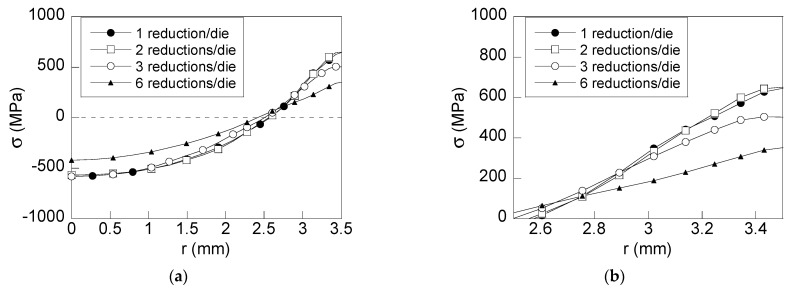
Radial distribution of hydrostatic stress: (**a**) general view; (**b**) detailed view of the important near-surface region of the wire.

**Figure 6:**
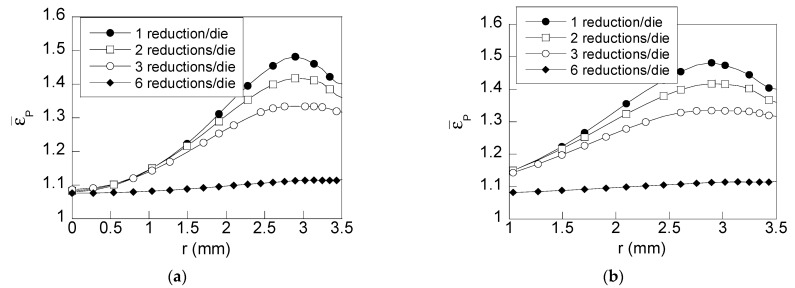
Radial distribution of equivalent plastic strain: (**a**) general view; (**b**) detailed view of the important near-surface region of the wire.

To estimate the improvement of the proposed wire drawing processes (Figure 1b–d), an analysis of the residual stress and strain states was developed. This analysis is focused, on one hand, on the variable representing the fracture in air of such wires: the *axial stress* *σ*_z_ (Figure 4) and, on the other hand, on the variables representing the stress and plastic strain states in the model of hydrogen diffusion assisted by stress and strain (Figure 5 and Figure 6), namely, *hydrostatic stress* (*σ*) and *equivalent plastic strain* (ε_p_). 

**Figure 7:**
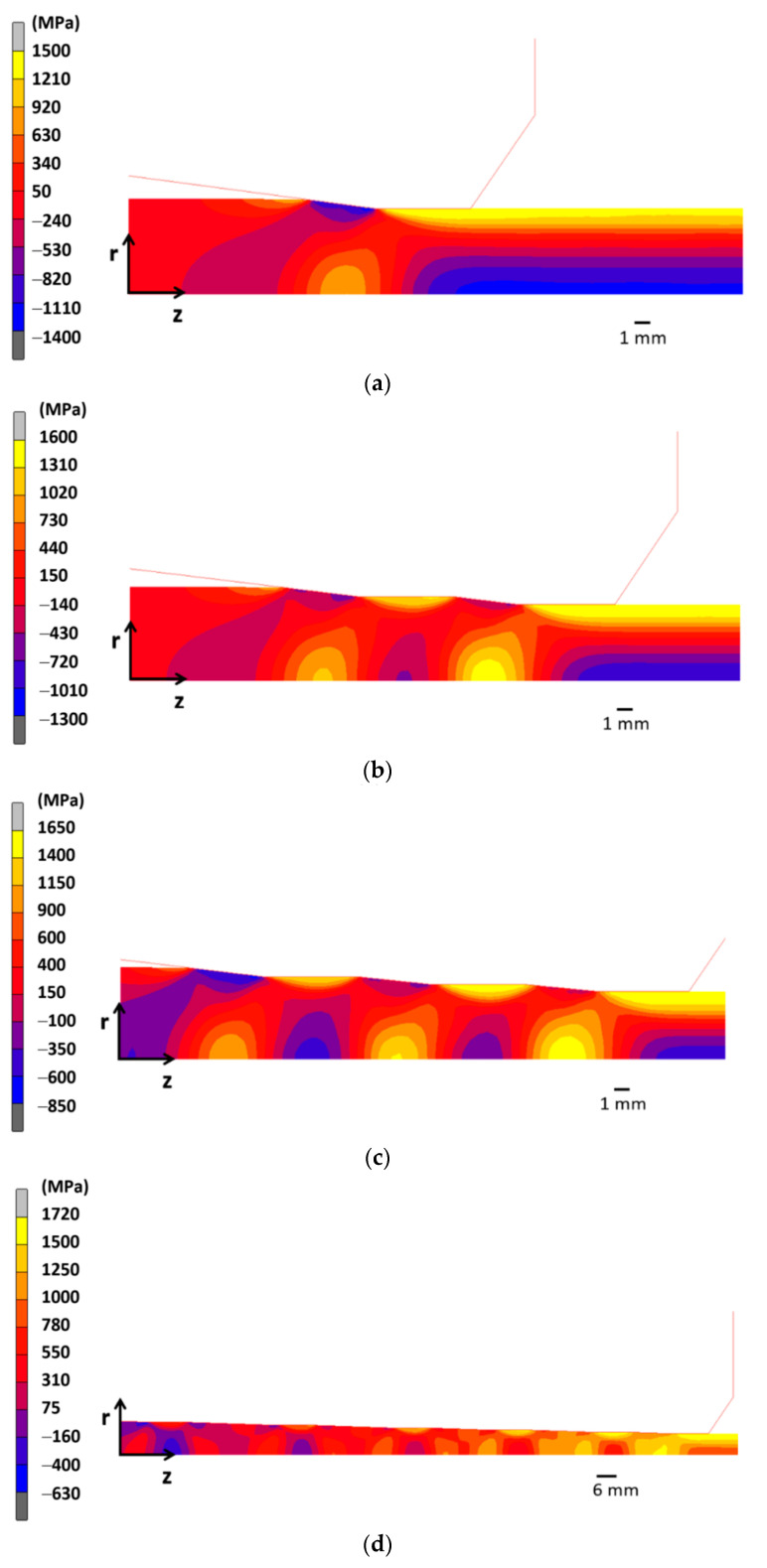
Axial stress fields during wire diameter reduction: (**a**) one reduction per die (conventional wire drawing), (**b**) two reductions per die, (**c**) three reductions per die, and (**d**) six reductions per die.

As Figure 4 shows, the main differences in the axial stress distributions are localized near to surface region (3 mm < *r* < 3.5 mm). However, the distributions are quite similar for deeper points of the wire (0 < *r* < 1 mm), as could be expected due to the surface-localized effect on stress fields during diameter reduction in wire drawing process (Figure 7). In addition, Figure 7 shows the axial stress fields generated during drawing obtained from the FE simulations of each wire drawing process considered. Thus, three zones can be observed: (i) before reduction zone, with the wire free of stress, (ii) within the reduction zone with compressive stresses at the contact die-wire and tensile stresses at the wire core, and finally, (iii) after passing the bearing length zone with a stress distribution with tensile stress at the wire surface and compressive stress at the wire core.

At the surface zone (Figure 4b), a reduction in axial residual stress is obtained (25%) with regard to the conventional drawing when the wire undergoes the drawing scheme C with three reductions per die (Figure 1c). The drawing scheme B with two reductions per die (Figure 1b) exhibits slight differences with regard to the conventional one. In the case of the drawing scheme D with six reductions per die (Figure 1d), the decrease in the axial stress at the wire surface is around 50% in relation to the conventional drawing (Figure 1a). In addition, a reduction in the stress state at the wire center (Figure 4a) is also achieved (42%). Thus, the wire drawing scheme *D* exhibits the most homogeneous residual stress distribution with significant reductions as high as 50% at the wire surface and the wire core.

The hydrostatic stress distributions obtained after the four considered drawing processes (residual state) look quite similar (Figure 5a) with tensile stress near the surface zone (2.5 mm < *r* < 3.5 mm) and compressive stress at inner points (0 < *r* < 2.5 mm). A reduction in hydrostatic stress at the surface is obtained in the wire drawn with three reductions per die (Figure 1c): 150 MPa, 22% with regard to the conventional process shown in Figure 1a. Nevertheless, the higher reduction is achieved by considering the multi-step die with six reductions per die (Figure 1d): 300 MPa, 45% regarding the conventional drawing process.

In the case of the equivalent plastic strain distribution (Figure 6), differences were obtained for each one of the wire drawing processes considered. These differences are localized over a wider zone (1 mm < *r* < 3.5 mm) than in the case of hydrostatic stresses. At the wire core surroundings (0 < *r* < 1 mm), an almost identical distribution is obtained with the exception of plastic strains generated after process D. According to obtained plastic strain distributions, the maximum difference is located at the position where the maximum value of ${\bar{\varepsilon }}_{P}$ is reached (*r* = 3 mm). At this position, the wire drawing process using multistep dies generates a lower equivalent plastic strain than the conventional wire drawing process (5% lower for the case using dies with two reductions per die (Figure 1b) and a 10% lower for the wire drawing process with three reductions per die (Figure 1c)). At the wire surface, a reduction in the plastic strains is also obtained (3% and 6% for the Process B and C, respectively). Nevertheless, the most interesting distribution is obtained by using a wire drawing chain D considering six reductions per die obtaining a highly uniform radial distribution of equivalent plastic strain. Thus, reductions in both, the maximum value of the distribution (25%) and plastic strains at the wire surface (20%), are reached for this case.

### 3.2. Hydrogen Embrittlement Susceptibility

**Figure 8:**
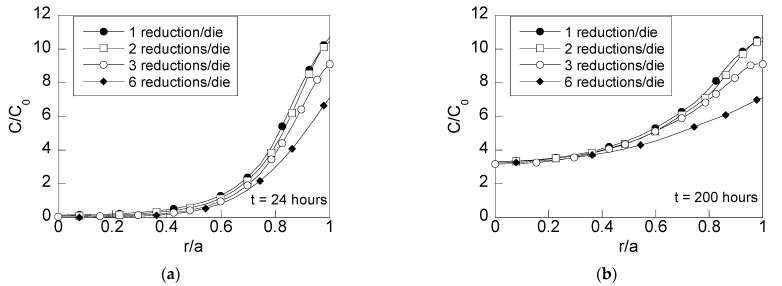
Radial distributions of relative concentrations of hydrogen (*C*/*C*_0_) as a function of the dimensionless radial coordinate *r*/*a*, at two different diffusion times: (**a**) *t* = 24 h and (**b**) 200 h.

The HE susceptibility can be estimated by analyzing the hydrogen accumulation in the wire for diverse times of exposure to the hydrogenating environment. This way, Figure 8 shows the radial distributions of hydrogen concentrations for short times of exposure (24 h) and long times of exposure (200 h, i.e., 8 days) in heavily drawn wires after each one of the drawing schedules previously depicted.

**Figure 9:**
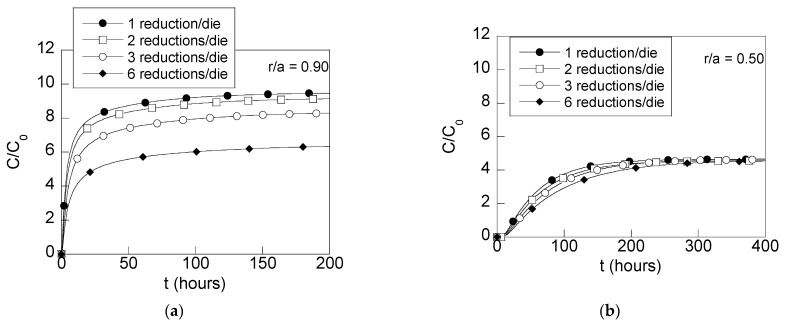
Time evolution of relative concentrations of hydrogen (*C*/*C*_0_) at two points placed at: (**a**) *r*/*a* = 0.90, (**b**) *r*/*a* = 0.50.

In addition, the time evolution of the hydrogen concentrations (Figure 9) was also obtained by FE simulations for two interesting places of the wire radius taking into account the stress fields (Figure 5 and Figure 6), i.e., (i) the depth *r*/*a* = 0.90 representing a potential damage zone near the surface, cf. [36] and (ii) the depth *r*/*a* = 0.50, *a* being the outer wire radius.

**Figure 10:**
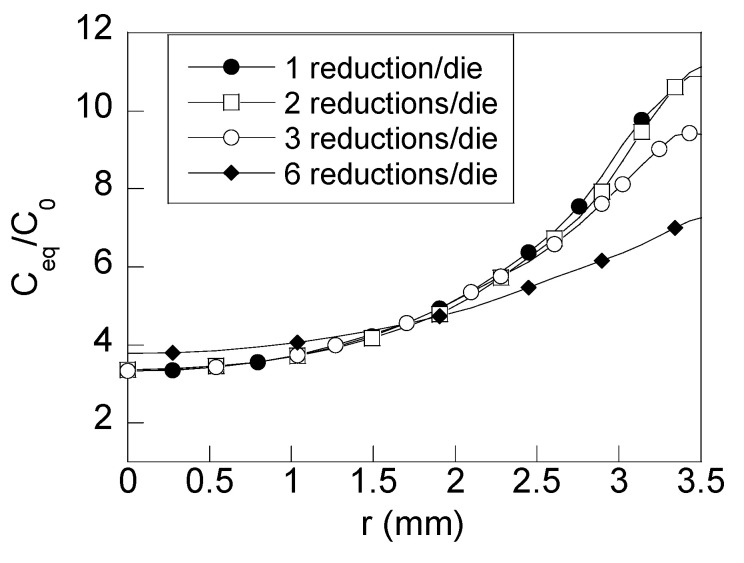
Radial distribution of hydrogen concentration for long times of exposure.

Finally, for assessing the implications of obtained results in the HE of drawn wires, an analysis of the long-time behaviour of the cold drawn wires exposed to a hydrogenating environment is carried out. Therefore, the hydrogen concentration distribution in the radial direction corresponding to the steady state was also obtained (Figure 10) after using Equation (3) considering both hydrostatic stress and equivalent plastic strain values given in Figure 5 and Figure 6. The plot of Figure 10 is linked to the infinite time of exposure to the hydrogenating source according to the steady-state solution from the mathematical point of view or, in other words, from the physical view point, with the hydrogen–metal system in thermodynamical equilibrium.

The distributions of hydrogen concentration revealed reductions in the hydrogen accumulation nearby the surface zone for short times of diffusion (uncomplete hydrogenation, i.e., when the hydrogen does not reach the wire center, Figure 8a). Thus, wires drawn with multistep dies of three reductions per die and six reductions per die exhibit a lower hydrogen concentration than a wire drawn with conventional dies (16% and 35%, respectively). For long times of exposure to the hydrogenating source (Figure 8b), such differences are still visible nearby the wire surface. However, as the depth from the wire surface increases, the hydrogen accumulation is similar for all the wire drawing cases considered. This way, at the wire center, the same hydrogen concentration is obtained for all the cases of study. The radial distribution of hydrogen concentration obtained for long times of exposure to the hydrogenating source for all the cases analyzed (Figure 8b) is similar to the ones given by the steady-state solution (Figure 10) for the differential Equation (3). An exception to this rule is observed at the zone close to the wire center of the wire drawn with process D (six reductions per die). Consequently, wires drawn with process D need more time to reach the steady state at inner points.

## 4. Discussion

On the basis of Equations (1)–(3) and considering the numerical results after FE modeling of the mechanical process of wire drawing (see Figure 5), the more significant reductions in the stress state are located nearby the wire surface for wires drawn with process C and D. In the particular case of the wire drawn process D (considering six reductions per die), a noticeable decrease in the hydrostatic stress state at the vicinity of the wire surface zone (45%) and at the wire core (30%) is observed. In addition, the inward gradient of hydrostatic stress (∂*σ*/∂*x*) is negative near the wire surface (2.8 mm < *r* < 3.5 mm), and consequently, it acts against hydrogen entry and diffusion. According to obtained results, the decrease in hydrostatic stress at the wire surface implies a reduction in the inward gradient of hydrostatic stress.

With regard to the second key variable (the equivalent plastic strain ε_P_), Figure 6 shows the results for the different drawing schemes. The upper curve is that of the conventional drawing process consisting of six steps of drawing (each of one associated with one die, i.e., one reduction per die), so that it is the reference curve. The wire drawing process considering two reductions per die (Figure 1b) produces a reduction of 29% in the in-going gradient of plastic strains (∂_εP_/∂*x*)_Γ_ at the surface (*r* = 3.5 mm) in relation to the conventional one (upper curve) at the same surface point. In the case of three reductions per die (Figure 1c), the reduction in the inward gradient of plastic strain at the surface (∂ _εP_/∂*x*)_Γ_ is as high as 78%. In the case of the wire drawing D, the reduction is the highest, reaching an almost horizontal distribution of plastic strain. Therefore, the inward gradient near surface zone (exposed to the hydrogenating source) is reduced up to a 95%. This way, the proposed wire drawing scheme causes slight reductions in the equivalent plastic strains and significant reductions in the gradient of equivalent plastic strains.

Taking into account the hydrogen concentration distributions shown in Figure 8, the effects of the reductions in the hydrostatic stress and equivalent plastic strains at the wire surface surroundings cause a reduction in hydrogen concentration of 16% for the wire drawn using dies with three reductions per die (process C) and 35% for wires obtained using the wire drawing process D with six reductions per die. In addition, the time needed for achieving the steady state for all the wire drawings is lower than 200 h, except for the wire drawing process D, which needed more time for increasing the concentration at the wire center vicinity. This effect can be clearly shown in the time evolution curves of the hydrogen concentration vs. time, appearing in Figure 9. Wires drawn with multistep dies of three and six reductions per die exhibit a slower accumulation of hydrogen. This can be easily observed paying attention to the slopes (d*C*/d*t*) of the curves in Figure 9. Thus, for points placed close to the surface (*r*/*a* = 0.90), different slopes are observed for the different drawing process, the faster one (higher slope, d*C*/d*t*) being the one corresponding to the conventional wire drawing process. Therefore, from these results, a sequence of hydrogen accumulation can be estimated as follows: as the number of reductions in the die is increased, the slope of the hydrogen time evolution curves is reduced, thereby resulting in a slower hydrogenation of the wire.

The hydrogenation at a point placed in the middle of the wire radius (Figure 9b) reveals in a clearer way the same sequence. Thus, the slope of the hydrogen time evolution curves is decreased as the number of reductions in the die is increased. As a result, the time for reaching the steady state for the wire drawn with six reductions per die is longer even when the similar maximum allowable hydrogen (*C*_eq_, i.e., the steady-state solution) is achieved for all the cases.

With regard to the long-time hydrogen concentration profile, Figure 10 shows that the concentration at the wire surface also exhibits reductions: 2% for the wire drawing process considering two reductions per die and 16% for the wire drawing process considering three reductions per die, with regard to the conventional wire drawing scheme with one reduction per die. In the case of the *process* D, the reduction in the hydrogen accumulation is the highest (35%). In addition, the hydrogen accumulation at the near surface zone is clearly lower in this case. This implies that hydrogen accumulation is reduced at the prospective damage zone observed in this type of manufactured prestressing steels [36] (*x* < 450 µm), and consequently, a lower susceptibility to HE should be expected for wires drawn using multi-step dies with six reductions per die.

Taking into account the aforesaid reductions in the driven forces for hydrogen diffusion, the wire drawn with *process* C (three reductions per die) or with *process* D (six reductions per die) exhibit a better behavior against HE than the wire drawn with *conventional wire drawing process* A (one reduction per die) because in both the C and D wires, the hydrogenation of wires will be slower than in the wire A (as indicated by the lower inward gradient of equivalent plastic strain in the C and D wires when compared to the A wire). In addition, for long times of exposure to the hydrogenating source, the final hydrogen content will be lower at the wire surface of C-D wires as well. This means that less hydrogen is potentially available for diffusing toward the inner points. Therefore, in general terms, *hydrogen damage in the wires drawn with wire drawing process C or D will be less probable to occur than in the wires drawn with conventional dies* (*process A*).

Finally, from obtained results regarding stress and strain reductions, the optimum wire drawing process is D (six reductions per die) because such a wire exhibits the lowest gradient of equivalent plastic strain (the distribution is almost horizontal near the surface zone or, in other words, the in-going gradient of hydrogen solubility is very low, Figure 6) and the lowest residual hydrostatic stress near the surface zone (Figure 5). In addition, the obtained reduction in the axial stress and plastic strain distributions also implies an improvement in the mechanical behavior (for instance, against crack initiation or fatigue crack propagation [11]) of the wire drawn with process C and D when compared to the conventional process A.

## 5. Conclusions

A new way of improving the mechanical behaviour against fracture in both inert and hydrogenating environments of cold drawn prestressing steel wires is proposed just considering a modification of the die geometry. This new design, which consists of applying to the wire several reductions per die (i.e., *multistep dies*), causes reductions in hydrostatic stress and equivalent plastic strain in both magnitude and inward gradients from the wire surface. According to the results, *the higher the number of reductions per die, the higher the reduction in both the magnitude and the inward gradient of plastic strains*. Thus, reductions up to 50% of the axial stress are obtained in both the wire surface and the wire core by using the wire drawing process with the highest number of reductions per die. In a similar way, reductions up to 45% of the hydrostatic stress are reached at the wire surface by using multi-step dies with six reductions per die. Regarding equivalent plastic strains, the reduction in the magnitude is low but the reduction in the inward gradient is noticeable reaching values as high as 95%. In other words, an almost plane distribution is obtained near the wire surface, thereby cancelling one of the driven forces for hydrogen diffusion. This way, on one hand, lowering the magnitude of hydrostatic stress and plastic strains implies reducing the hydrogen content at the zone of prospective micro-damage by HE, and on the other hand, reducing the inward equivalent plastic strain gradient means decreasing the driven force that enhances hydrogen diffusion from hydrogenating surface toward prospective fracture points.

Thus, by applying the proposed changes in die geometry (*several reductions per die*), cold drawn wires would exhibit a lower susceptibility to hydrogen embrittlement than that of the drawn wires with conventional process (one reduction per die). As a final conclusion, according to the obtained results, the optimum wire drawing scheme corresponds to the process where more reductions per die were considered.

## Data Availability

Not applicable.
